# Light-Switchable Membrane Permeability in Giant Unilamellar Vesicles

**DOI:** 10.3390/pharmaceutics14122777

**Published:** 2022-12-12

**Authors:** Paola Albanese, Simone Cataldini, Chloe Z.-J. Ren, Nadia Valletti, Jlenia Brunetti, Jack L.-Y. Chen, Federico Rossi

**Affiliations:** 1 Department of Earth, Environmental & Physical Sciences, University of Siena, Pian Mantellini 44, 53100 Siena, Italy; 2 Department of Biotechnology, Chemistry and Pharmaceutical Sciences, University of Siena, Via Aldo Moro, 53100 Siena, Italy; 3 Centre for Biomedical and Chemical Sciences, School of Science, Auckland University of Technology, Auckland 1142, New Zealand; 4 MedBiotech Hub and Competence Center, Department of Medical Biotechnologies, University of Siena, Via Aldo Moro 2, 53100 Siena, Italy; 5 The MacDiarmid Institute for Advanced Materials and Nanotechnology, Victoria University of Wellington, Wellington 6140, New Zealand

**Keywords:** giant vesicles, stimulus-responsive membranes, photoswitchable amphiphiles, azobenzene, light-responsive vesicles, smart materials, systems chemistry, targeted drug delivery

## Abstract

In this work, giant unilamellar vesicles (GUVs) were synthesized by blending the natural phospholipid 1-palmitoyl-2-oleoyl-*sn*-glycero-3-phosphocholine (POPC) with a photoswitchable amphiphile (**1**) that undergoes photoisomerization upon irradiation with UV-A (*E* to *Z*) and blue (*Z* to *E*) light. The mixed vesicles showed marked changes in behavior in response to UV light, including changes in morphology and the opening of pores. The fine control of membrane permeability with consequent cargo release could be attained by modulating either the UV irradiation intensity or the membrane composition. As a proof of concept, the photocontrolled release of sucrose from mixed GUVs is demonstrated using microscopy (phase contrast) and confocal studies. The permeability of the GUVs to sucrose could be increased to ~4 × 10^–2^ μm/s when the system was illuminated by UV light. With respect to previously reported systems (entirely composed of synthetic amphiphiles), our findings demonstrate the potential of photosensitive GUVs that are mainly composed of natural lipids to be used in medical and biomedical applications, such as targeted drug delivery and localized topical treatments.

## 1. Introduction

**Figure 1:**
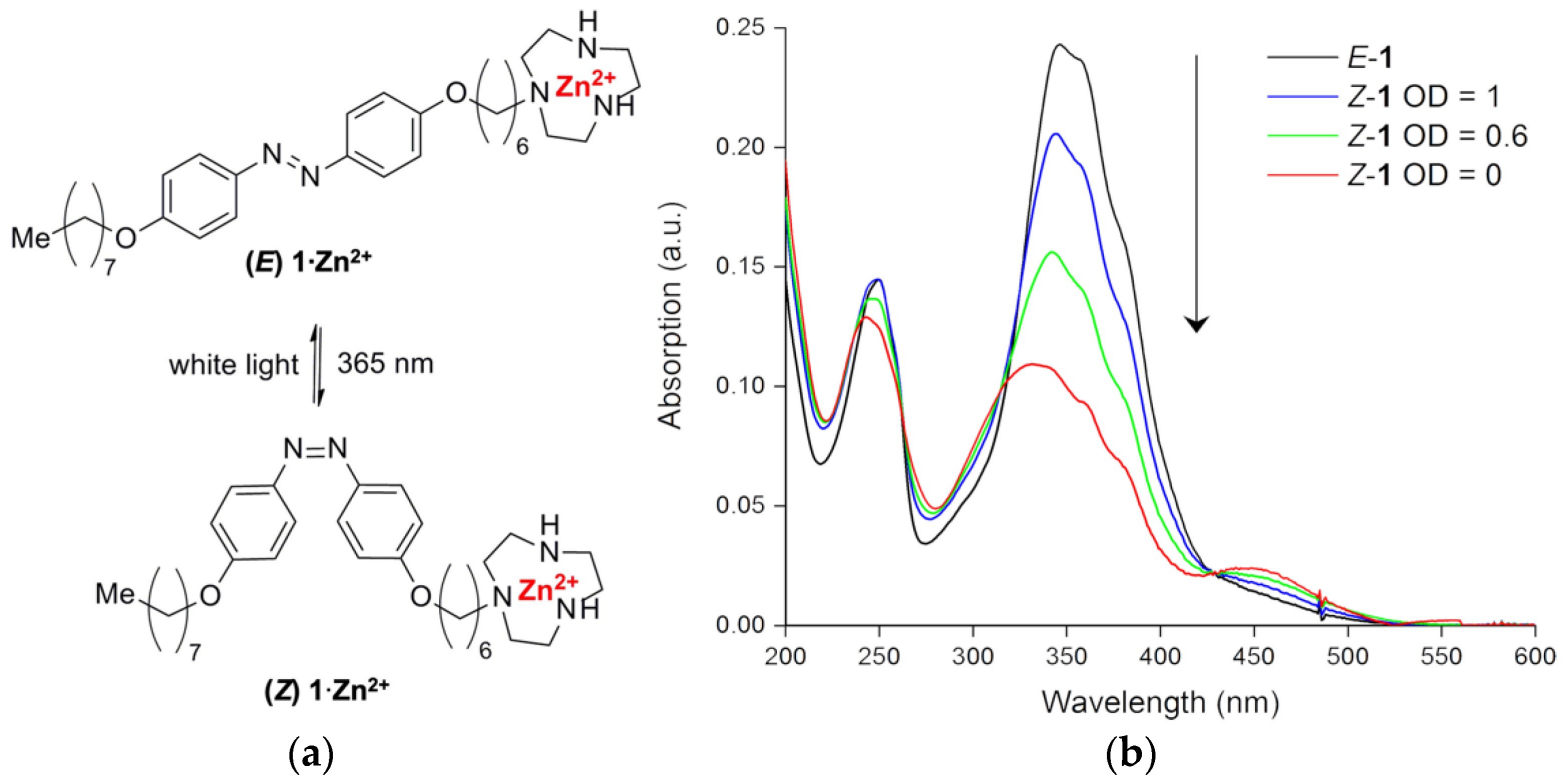
Photoinduced isomerization of the amphiphilic molecule **1**·Zn^2+^. (**a**) Chemical structure and geometry of the *E*- and *Z*-**1** isomers; (**b**) absorption spectra of **1** (150 µM) in HEPES (5 mM) buffered solution at pH 7. The downward arrow indicates a progressive decrease in irradiation shielding.

Synthetic biology and systems chemistry are fast-growing disciplines that aim to engineer synthetic systems with features that mimic the complexity of life [1,2,3,4,5,6]. The ultimate goal is the construction of artificial (minimal) cells able to accomplish complex tasks in a controlled fashion. Thus far, research in this area has been successfully utilized for the design of nano- and micro-objects (e.g., particles, micelles, vesicles) which have numerous applications as smart materials, in targeted drug delivery, and in biocompatible devices [7,8,9,10,11]. In this context, the development of stimulus-responsive materials and the dynamic components needed to fabricate them are attracting the attention of numerous researchers due to their importance for the construction of objects that can interact with the environment and perform autonomous actions in response to external inputs [12,13,14,15]. Notable examples include drug carriers (e.g., liposomes, capsules) that can sense the physicochemical characteristics (e.g., pH, temperature) of different organs, tissues, and/or cells, and release their cargo only when the target is reached [10,16,17,18,19,20]. Light is one of the most investigated stimuli for controlling the response of smart materials; therefore, the development of a library of photosensitive molecules would help in the rational design of micro- and nanodevices able to exhibit responsive behavior. In particular, there is growing interest in the field of photopharmacology, which uses light to modulate the pharmacokinetics and pharmacodynamics of biologically active compounds by introducing molecular photoswitches in their structure. Among the available synthetic photocontrol mechanisms, azobenzenes are the most studied candidates to be incorporated into a light-responsive medical device. Their success can be ascribed to the favorable photophysical properties and the absence of phototoxic side effects, which were demonstrated in several in vitro [21,22] and in vivo [23] studies. Molecules containing the azobenzene group can change their geometry from trans (*E* form, thermodynamically more stable) to cis (*Z* form) when irradiated by UV light (*λ* ~365 nm) and switch back to their original form via thermal relaxation or if irradiated with blue (*λ* ~465 nm) or visible light, as shown in Figure 1a.

So far, azobenzene moieties have been successfully integrated into (i) receptors and ion channels [23,24], (ii) transporters and ion pumps [25], (iii) enzymatic ligands and inhibitors [25], (iv) DNA [26], (v) antimicrobics [27], (vi) prodrugs and drug delivery systems [28], allowing for their remote photoactivation and/or photomodulation with high spatial and temporal resolutions. Despite the biomedical applications of synthetic photoswitches still being in their preliminary stages, their efficacy has been demonstrated at the cellular level and, in some cases, on animal models, for the treatment of microbial infection, diabetes, cancer, pain, and blindness, providing evidence of their biocompatibility and for promising therapeutical applications in the near future [25].

In this work, we are interested in photoresponsive vesicles and liposomes that could be employed in applications involving cargo release. Photoswitchable amphiphilic molecules have been employed for the doping of mixed giant unilamellar vesicles (GUVs) composed of natural lipids, which burst and release their cargo following illumination with UV light [29]. More recently, a finer control of permeability was attained by forming GUVs entirely comprising photosensitive phospholipids and containing a phosphatidylcholine head group and one or two acyl chains containing an azobenzene [30]. The trans form of the diazobenzene confers to the lipid a linear shape that favors membrane self-assembly. When irradiated with UV light, the acyl chain containing the azobenzene group assumes an L shape that destabilizes the membrane and modifies its packing parameter. This allows for the precise control of the shape of the GUVs and their permeability [31,32,33,34,35].

In this work, we describe a new type of photoresponsive GUV prepared by blending the 1-palmitoyl-2-oleoyl-*sn*-glycero-3-phosphocholine (POPC) phospholipid and a photoswitchable amphiphile (**1**, Figure 1a), recently synthesized by us [36]. Phosphatidylcholine lipids represent one of the major components of eukaryotic cellular membranes; among them, POPC is the most abundant [37,38]. For this reason, POPC-based vesicles are widely studied as model membranes in the biophysical and biomedical fields, and have been safely employed as biocompatible carriers for in vivo and in vitro drug delivery applications [39,40,41,42,43]. We show that fine control of cargo release is attainable upon light stimulation of GUVs that are composed mainly of natural lipids. The modulation of bilayer permeability, with consequent control of release kinetics, can be achieved by modifying either the UV irradiation intensity or the membrane composition.

## 2. Materials and Methods

### 2.1. Chemical Synthesis of Photoswitchable Compound ***1***

**Scheme 1:**
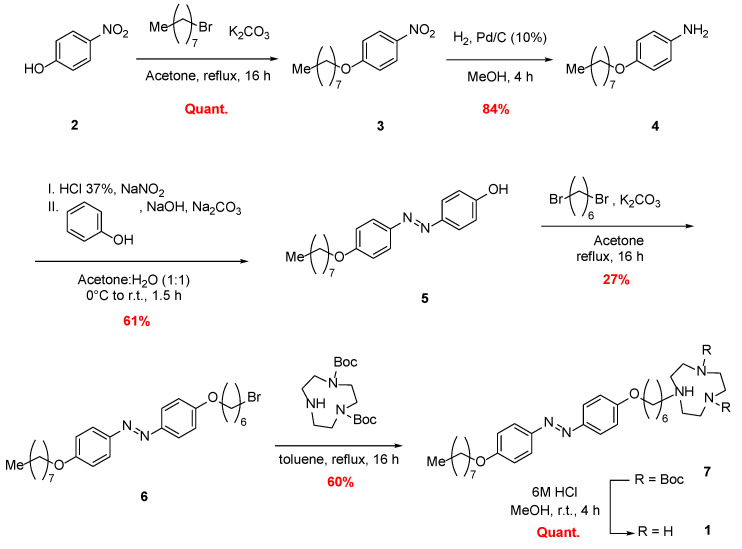
Synthetic route to photoswitchable amphiphile **1**.

The synthesis of photoswitchable amphiphile **1** was accomplished by exploiting a synthetic route beginning with the alkylation of 4-nitrophenol (**2**) with 1-bromooctane (Scheme 1). The photoswitchable diazobenzene moiety was installed by reduction of the nitro group followed by diazotisation, and subsequent diazo coupling with phenol as the nucleophile. The alkylation of Compound **5** with 1,6-dibromohexane afforded bromide intermediate **6**, which was subjected to alkylation with di-Boc-protected triazacyclononane (TACN) to afford Compound **7**. Lastly, the acid-promoted removal of Boc-protecting groups afforded the desired photoswitchable amphiphile **1** as an HCl salt [36,44].

To increase the amphiphilicity of Compound **1**, an equimolar amount of ZnCl_2_ was added, since Zn^2+^ possesses a very high binding constant with TACN. The resulting stable 1·Zn^2+^ complex was the photoswitchable amphiphile that was incorporated into the GUVs.

### 2.2. Optical Spectroscopy

The *E*,*Z*-photoisomerization of amphiphile **1** was investigated spectrophotometrically in bulk solution as described in [36]. The configurational switch in the molecule occurs with a change in the absorption spectrum, with the *E* and *Z* forms possessing absorption peaks with $\lambda_{max}$ = 350 and 330 nm, respectively. Samples of **1** (150 µM) in a buffered solution of HEPES-KOH (5 mM) at pH 7 were prepared in a quartz cuvette (Hellma 110-1-40, Sigma Aldrich, St. Louis, MI, USA) and irradiated using a 100 W mercury short-arc lamp (HBO Osram, Wilmington, MA, USA, spectral irradiance ~100 mW/m^2^/nm) with bandpass filters to provide irradiation at $\lambda_{max}$ = 365 nm. Samples were irradiated for 30 s, and absorption spectra were recorded with an Agilent 8453 spectrophotometer (Agilent Technologies, Inc., Santa Clara, CA, USA). The irradiation intensity was tuned by interposing neutral density filters (Thorlabs NDL-25S-2, Newton, NJ, USA) with increasing optical density (OD) between the specimen and the UV light source. Specifically, OD = 0.6, OD = 1, and OD = 2 were tested, which corresponded to 30%, 12%, and 2% of the light transmission at 365 nm, respectively.

### 2.3. Preparation of Phospholipid Giant Unilamellar Vesicles

Giant unilamellar vesicles were prepared following the phase-transfer method [45] with some modifications [46,47,48,49,50]. The method requires the preparation of three solutions: the organic phase (OP), the outer solution (OS), and the inner solution (IS). The OP consisted of mineral oil (Sigma-Aldrich, St. Louis, MO, USA, #M5904) in which the membrane building blocks were dispersed. As the amphiphilic molecule, phospholipid POPC (Lipoid GmbH, Steinhausen, Switzerland) was used in a concentration range of 0.150–0.300 mM. Cholesterol (Sigma-Aldrich, St. Louis, MO, USA, #57-88-5) was added to achieve a final concentration of 40 mol% with respect to the lipid. The IS ([sucrose] = 200 mM and [HEPES-KOH] = 5 mM pH 7) represents the aqueous solution that was to be confined within the GUVs, while the OS ([glucose] = 200 mM, [HEPES-KOH] = 5 mM pH 7) represents the buffer within which the GUVs were dispersed after their formation.

As in a typical preparation, 300 µL of the OP was stratified on top of 500 µL of OS in a 1.5 mL Eppendorf tube and allowed to rest for 15–30 min. During the incubation time, phospholipids spontaneously saturate the two-liquid interface of the biphasic system, exposing the hydrophilic heads towards the aqueous lower solution and the hydrophobic tails to the upper oil phase. In a new 1.5 mL Eppendorf tube, 20 µL of inner solution was added to 600 µL of OP and emulsified by manual pipetting. The obtained phospholipid-stabilized water-in-oil emulsion was gently overlain on top of the biphasic system, and the sample was centrifuged for 10 min at 2500× *g* at room temperature. The centrifugation forces the migration of the emulsion into the lower OS. By crossing the liquid–liquid interface of the biphasic system, the stabilized water droplets acquire a second layer of phospholipids, forming GUVs that accumulate as a pellet at the bottom of the tube. The GUV pellet was collected by aspiration with a micropipette into a final volume of 50 µL. The giant lipid vesicles were washed twice and resuspended with fresh OS.

Equimolar sugars with different molecular weights were included in the IS and OS to obtain a density gradient between the two aqueous solutions. The gradient promotes the transfer of water droplets from the emulsion across the w/o interface and enhances phase contrast during the microscopy experiments. To confer photoresponsive properties, we integrated **1**·Zn^2+^ into the phospholipid membrane of the vesicles by including *E-***1**·Zn^2+^ at the desired concentration into the solution containing the GUVs.

### 2.4. Observations under Microscopy of Light-Triggered GUV Shape Transitions

Photoresponsive GUVs were prepared as described and functionalized with [**1**·Zn^2+^] = 75 µM (POPC: **1**·Zn^2+^ = 2:1). UV-induced membrane deformations were imaged by means of a Leica SP8 inverted scanning confocal microscope using a 40× oil immersion objective (HC PL APO 40×/1,10 W CORR CS2). For visualization, GUV membranes were stained with 5 µM of a Nile red-derived fluorescent dye ($\lambda_{ex}$ = 515 nm, $\lambda_{em}$ = 550–700 nm), synthesized as described in [51]. For each experiment, 50 µL of the vesicle solution was placed on a customized glass microscopy chamber. The images of the shape transitions were acquired during concomitant sample irradiation with 515 and 405 nm laser lines. Deformations soon reverted when the 405 nm irradiation was interrupted, demonstrating that the photosensitive molecule spontaneously relaxed back to the more stable *E*-isomer. Digital videotapes were recorded at 1 fps with a CMOS camera (PIXELINK, Ottawa, ON, Canada, PL-D755CU). Control samples were prepared in the absence of photoswitchable compound **1**·Zn^2+^.

### 2.5. Cargo-Release Experiments

Cargo-release experiments were performed using phase-contrast microscopy. GUVs were prepared as described above ([POPC] = 0.3 mM) and resuspended into a final volume of 120 µL of fresh outer solution. The sample was split into 20 µL aliquots, each one incubated in the dark with a different *E*-**1**·Zn^2+^ concentration as follows: [**1**·Zn^2+^] = 0, 30, 75, 150, 240, and 300 µM. Following 5 min of incubation, the samples were placed in a microscopy chamber and analyzed. For sample imaging, an ORMATEK TL-INV 100 microscope was used with a 20× objective (Plan 20×/0.4) and phase-contrast filter. Images were recorded every 1 s with a CMOS camera (PIXELINK PL-D755CU) in visible mode with concomitant UV illumination ($\lambda_{max}$ = 365 nm). Control samples were prepared in the absence of the photoswitchable molecule. The attenuation of UV intensity was achieved by means of a step-variable neutral density filter (Thorlabs NDL-25S-2). Images were analyzed using ImageJ software, version 1.8.0.

## 3. Results and Discussion

The photoswitchable properties of amphiphilic molecule **1** were first examined in aqueous solution buffered at pH = 7. The *E*-*Z* photoisomerization of the azobenzene group was driven by UV light (365 nm, *E* to *Z*) and blue light (475 nm, *Z* to *E*), as shown in Figure 1a. Under standard conditions, when exposed to natural light, the *E* form is thermodynamically favored and is the most abundant species. The spectrum reported in Figure 1b (black line) shows, in fact, a characteristic single absorption maximum at 350 nm. Following irradiation of the sample with light filtered at 365 nm (spectral irradiance ~100 mW/m^2^/nm) for 30 s, the absorption of UV light induces the isomerization of the *E*-diazobenzene to the bent *Z* form, with a concomitant blue shift of the absorption maximum to 330 nm and the appearance of a pronounced shoulder at λ = 450 nm (red trace in Figure 1b). Quantitative switching back to the *E* isomer could be achieved with either irradiation with blue light or spontaneous thermal relaxation over time. The influence of irradiation intensity on the photoswitching behavior was examined by adding a neutral density filter between the light source and the UV bandpass filter, and modifying the optical densities along the light path. Figure 1b shows that, following a fixed irradiation time, the amount of *E-***1**·Zn^2+^ converted into the *Z* form was clearly dependent on the intensity of the irradiation. The conversion yield was ~60% for a filter with optical density (OD) = 0.6 (30% of the light transmission at 365 nm, green trace in Figure 1b) and ~30% for an OD = 1.0 (12% of the light transmission, blue trace in Figure 1b), while it became inappreciable when OD = 2 (2% of the light transmission).

Following confirmation of the switching ability of amphiphilic **1**·Zn^2+^, photoresponsive GUVs were prepared using the phase-transfer method. For all experiments, the membrane was composed of 1-palmitoyl-2-oleoyl-*sn*-glycero-3-phosphocholine (POPC) and 40% of the molar fraction of cholesterol, with the latter chosen to increase the rigidity of the membrane and enhance the effect of the light-induced modifications. The photoswitchable molecule was delivered at the desired concentration to the membrane by infusing a **1**·Zn^2+^ solution directly into the dispersing medium of the preformed GUVs. The assumption was that **1**·Zn^2+^ spontaneously intercalated into the bilayer of the vesicle membrane to shield its hydrophobic chain from the surrounding aqueous environment.

In a typical experiment, after a fixed infusion time (5 min), 50 µL of sample would be placed in a multiwell glass plate and covered to prevent evaporation. The dynamics of the system was monitored with confocal microscopy and, for better visualization, the GUVs were stained with a Nile red (NR)-derived amphiphilic fluorophore [51] that absorbs and emits in a wavelength range far from what we used to stimulate the *E–Z* transition of the azobenzene group (λ_ex_ = 515 nm, λ_em_ = 550–700 nm).

**Figure 2:**
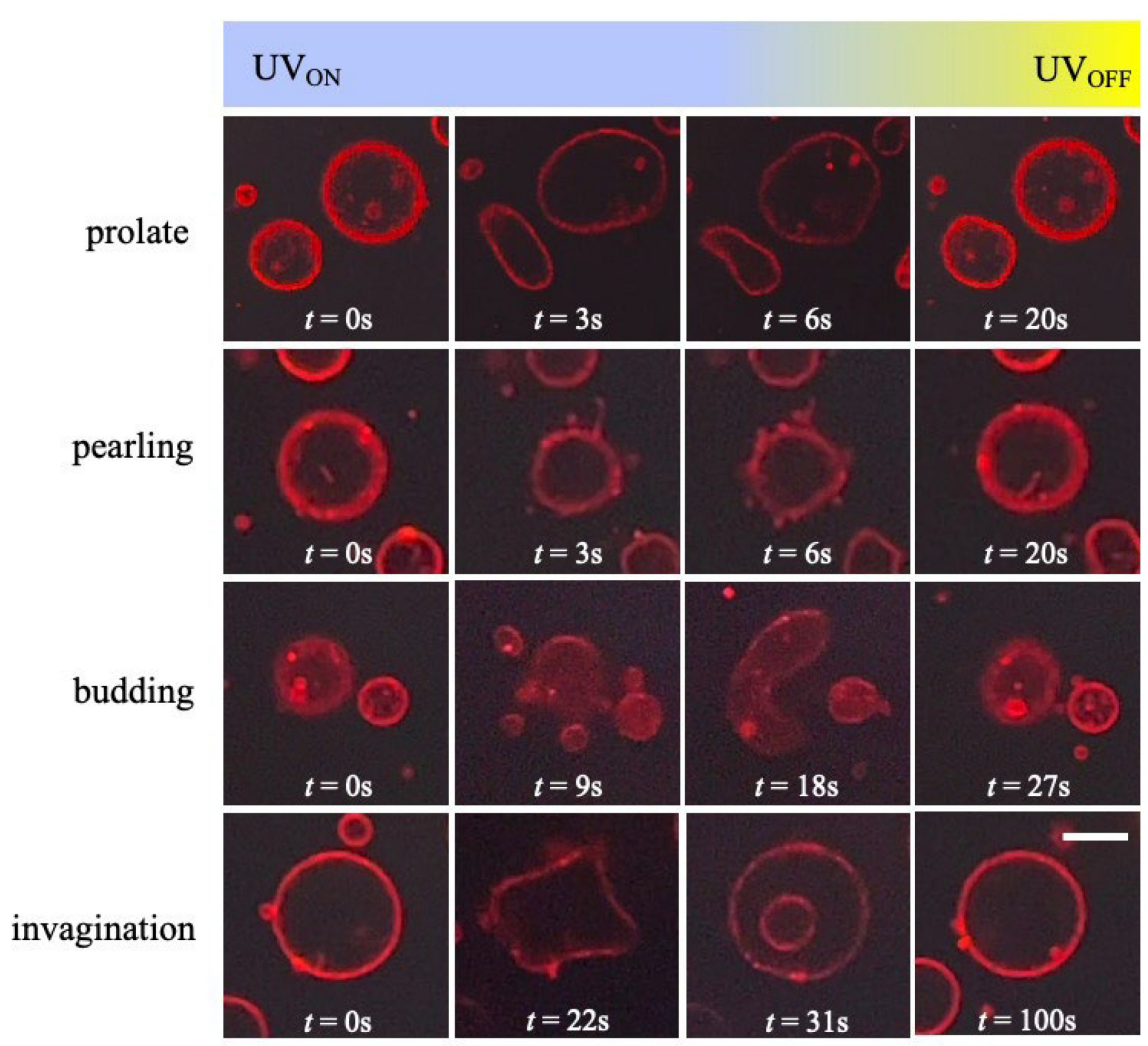
Confocal images showing light-triggered GUV shape transitions in the presence of **1**·Zn^2+^ (75 µM). The GUV membrane was stained with NR-derived fluorophore (5 µM; $\lambda_{ex}$ = 515 nm, $\lambda_{em}$ = 550–700 nm) while *E*-*Z*-**1**·Zn^2+^ photoisomerization was controlled by switching the 405 nm UV laser line on and off. The first and the last frames of each line show the GUVs before and after UV illumination, respectively. White scale bars = 15 µm.

Analysis revealed that the presence of **1**·Zn^2+^ allowed for UV-triggered shape deformations in preformed spherical vesicles over a wide range of concentrations. As an example, Figure 2 shows a collection of frequently observed deformations detected in a population of GUVs when [**1**·Zn^2+^] = 75 µM. Commonly, membrane deformations begin immediately after the UV light is switched on, and the types of observed changes include transitions from spherical to prolate shapes, pearling, budding, and, less frequently, invaginations. In all cases, the vesicles regained their original shapes a few seconds after the UV irradiation had been interrupted due to the spontaneous thermal relaxation of *Z*-**1**·Zn^2+^ to the more stable *E*-configuration. Continuous cycling between the spherical and modified shapes could be achieved by switching the UV laser line on and off. We performed up to 10 cycles without any loss in light responsiveness. The proportion of photoresponsive GUVs that had undergone light-induced shape modifications was consistently between 70% and 100%. A complete absence of shape transitions was observed in the control sample, which was identically prepared, but without the incorporation of **1**·Zn^2+^.

To understand how the *E–Z* transition impacts the shape of the GUV population, we can take into account area difference elasticity (ADE) theory [52,53], which considers a vesicle to be an elastic membrane, and its equilibrium shape can be found by the minimization of Helfrich energy [54,55,56]. According to ADE theory, a spherical vesicle undergoes a shape transition when its volume decreases, and, at the same time, the area difference between the outer and inner leaflets of the bilayer, Δ*a* = *a*_out_ − *a*_in_, diverges from what is preferred (i.e., that of a sphere). The transition to a prolate sphere, and pearling and budding dynamics are typical of an increase in the difference in the area between the outer and inner leaflets [57,58,59,60,61], indicating that **1**·Zn^2+^ is mainly located in the outer leaflet of the GUVs. In fact, the *E* form has a linear geometry that favors the intercalation of the azomolecule parallel to the POPC phospholipids. Following a switch to the *Z* form, the lateral size of its hydrophobic chain increases, causing an increase in the surface area of the outer leaflet. The less frequent invagination behavior likely occurs when **1**·Zn^2+^ is located in the inner leaflet of the membrane, which causes a decrease in the area difference typical for this shape transition [55,58].

Given the employed preparation method to obtain the photoresponsive GUVs, it was difficult to precisely control the location of the azobenzene-based amphiphilic molecules within the membranes. Therefore, GUVs belonging to the same population could show contrasting behaviors. However, what is clear from the experiments is the fact that, when **1**·Zn^2+^ is in the *Z* geometry, the vesicles lose a part of their inner content. After the incorporation of the amphiphile, its counterion (Cl^–^) might generate an outer hypertonic solution; however, the low concentration of the ion (corresponding to Δ[Osm] = 6 × 10^–4^ M in the most concentrated experiments) is unlikely to cause a deflation that, according to ADE theory, has to be at least ~20% for the observed equilibrium shapes. A more plausible explanation would be that the transition from *E* to *Z* triggers the formation of pores and defects in the GUV membrane, as previously observed in photoresponsive vesicles made entirely of azobenzene-based phospholipids [33]. This characteristic could thus be exploited for delivery applications in medical imaging or drug delivery, as previously indicated for azolipid-based nano- and giant vesicles [31,33,62].

Therefore, having shown that the photoswitchable amphiphilic **1**·Zn^2+^ can modify bilayer order and dynamics in a light-responsive way, we proceeded to investigate how photoisomerization could alter the membrane permeability towards small water-soluble molecules. In order to understand the changes in the composition of the GUV lumen, we monitored the samples in real time by using a stereomicroscope equipped with a phase-contrast (PC) setup. The PC technique allows for visualizing the phase shift that occurs when light passes through a transparent object having a different optical density by converting the phase shift into brightness gradients according to the refractive index of the material. Therefore, in a grayscale image, denser zones appear to be darker in the image, while less dense areas appear to be brighter.

**Figure 3:**
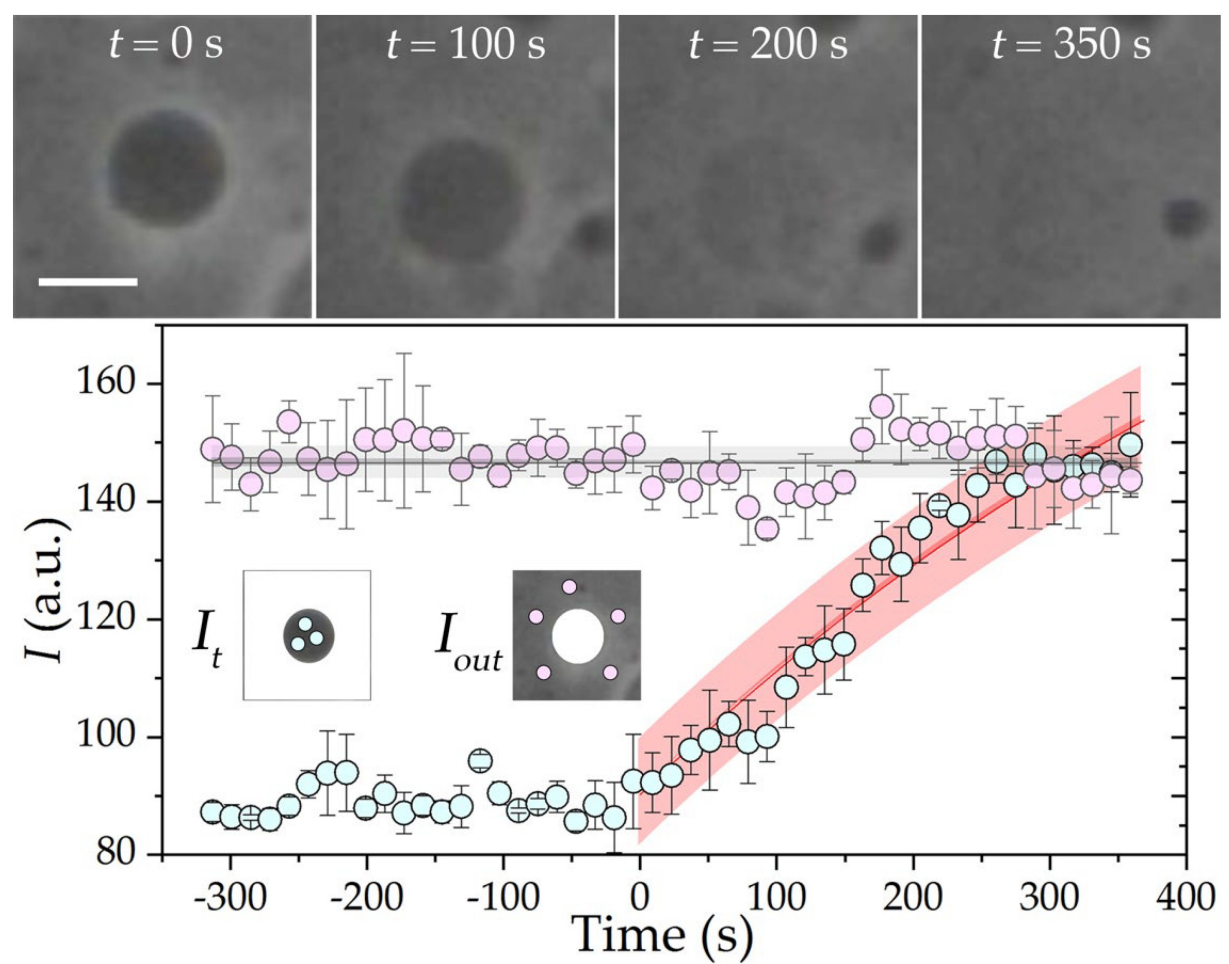
(**top** panel) Phase-contrast imaging of a cargo release experiment with [**1**·Zn^2+^] = 150 µM, [sucrose]_in_ = 200 mM, and [glucose]_out_ = 200 mM. The UV lamp was switched on at *t* = 0; white bar scales = 10 µm. (**bottom** panel) Kinetic analysis of the intensity extracted from the experimental micrographs. Cyan circles, with the associated standard deviation, represent the average *I_t_* extracted from the ROIs depicted in the left inset. The red trace represents the fitting to Equation (3) with the following parameters: *I_out_* = 146.2, *I*_0_ = 90.23 and *r* = 3.98 µm; red bands represent the 95% fitting confidence. Pink circles, with the associated standard deviation, represent the average *I_out_* extracted from the ROIs depicted in the right inset. The gray trace represents the best linear fitting with slope = 0 and intercept 146.2; gray bands represent the 95% fitting confidence.

In our experiments, GUVs were filled with 200 mM sucrose buffer (ρ = 1.0238 g/cm^3^) and resuspended in an isosmotic glucose solution (ρ = 1.0116 g/cm^3^). The different densities of the two sugars rendered the inner solution of vesicles darker than the surrounding aqueous medium, as shown in Figure 3 (top panel). In general, given their polar structure and their relatively large sizes, the two sugars are virtually impermeable to the POPC-based membrane (the permeability of sucrose was estimated to be ~10^–6^–10^–5^ μm/s depending on the membrane composition [63,64]), and in the absence of the photoswitchable molecule, the contrast is preserved indefinitely in time. However, with **1**·Zn^2+^ present in the membrane, we expect that the light-induced switch from the *E* to the *Z* form increases the permeability of the bilayer towards the sugars, thus allowing for mass exchange between the lumen of the vesicle and the surrounding solution. The diffusion of the sugars driven by the initial concentration gradient eventually equilibrates the distribution of the solutes inside and outside the GUVs with a consequent loss of the phase contrast. In other words, we expect that, upon illumination with UV light and the successful release of the GUV cargo, the lumen of the vesicle would progressively become brighter until it reached the same gray level as that of the outer solution.

We explored different compositions of the GUV membrane with [**1**·Zn^2+^] in the range of 0–300 µM to understand how the concentration of the photoswitchable amphiphile influences the release mechanism. For this experiment, the phospholipid concentration was increased to [POPC] = 0.3 mM in order to maximize the yield of the GUVs and to monitor a larger population. As an example, Figure 3 reports the release kinetics of GUVs formed when [**1**·Zn^2+^] = 150 µM. At the beginning of the experiment, the lumen of the GUV was darker than the surrounding solution, but following the irradiation of the sample with UV light (*t* = 0), a gradual increase in the inner brightness was observed until the contrast between the lumen and the dispersing solution disappeared (t ~ 6 min), as shown in the top panels of Figure 3. The cyan timeseries in the bottom panel of Figure 3 depicts the average lumen intensity extracted from 3 regions of interest (ROI, cyan circles in the left inset). The release dynamics followed an exponential trend (red line), as expected for the passive diffusion of small molecules through a semipermeable membrane. In fact, the flux of the sucrose (*J = dn/dt* × *a^–1^*, where *n* is the moles of sucrose) is proportional to the permeability of the sucrose (*P*), and to the difference in the inner and outer concentrations of the sugar (*J* = *P* × Δ*C*), which can be described with the following first-order differential equation:

(1) $$\frac{dC_{in}}{dt}=-\frac{aP(C_{in}-C_{out})}{V_{in}}$$

where *C_in_* is the concentration of sucrose in the GUV lumen, *C_out_* is the concentration of sucrose in the outer solution, and *V_in_* is the inner volume of the GUV. The same equation is valid for the inflow into the vesicle of glucose from the surrounding solution. The total inner volume of the GUVs in the specimen ($V_{in}^{tot}=\sum_{n}V_{in}∼6\times 10^{-3}$μL, with *n* ~ 30,000 and $V_{in}∼2\times 10^{-7}$μL) was much lower than the volume of the outer solution (*V_out_* = 20 μL). Therefore, *C_out_* could be considered to be constant with respect to both glucose (200 mM) and sucrose (0 mM). Due to the backlight scattering of the specimen and the contribution of the out-of-focus light from outside the focal plane, the PC technique did not allow for a quantitative determination of the concentration of the solutes in the sample. However, in the case of a spherical GUV, a semiquantitative approach allows for inferring precise values for the permeability even without a calibration curve for the image intensity. The difference in the contrast between the lumen of a GUV and the background could be normalized to obtain an equation for *P,* which is independent from the diffusion of light in stereomicroscopes [64,65,66]. Equation (1) allows for analytic solutions, and, to simplify the calculations, it is more convenient to consider the counterflux of glucose, which replaces the outflow of sucrose to conserve the osmotic balance. Hence, the solution of Equation (1), with initial conditions *C_in_* (*t* = 0) = 0 and *C_out_* kept constant, reads

(2) $$C_{in}(t)=C_{out}-C_{out}e^{-\frac{3P}{r}t}$$

with *C_in_*(*t*) being the variation in the concentration of glucose in time, and *r* the radius of the GUV. The out-of-focus backlight depends only on the radius of the GUVs and not on the concentration of the solutes; therefore, Equation (2) can be rewritten in terms of the gray level intensity (*I*) of the image as follows [65,66]:

(3) $$I_{t}=I_{out}\left(1+\frac{I_{0}}{I_{out}}-e^{-\frac{3P}{r}t}\right)$$

where *I_t_* is the variation of the intensity of the GUV lumen over time, *I_0_* is the intensity of the GUV lumen at *t* = 0, and *I_out_* is the intensity of the outer solution. The value for *I_out_* was extracted as the intercept of the linear fitting of the average intensity of the background (pink circles in the bottom panel of Figure 3) sampled at 5 different locations (right inset). Lastly, the average intensity of the lumen was fitted to Equation (3) to find the value of *P* (more details in the caption of Figure 3). Following this approach and extending the analysis to a statistically significant population of GUVs (~40), the average permeability of glucose (sucrose) when **1**·Zn^2+^ is in the *Z* form was ~2.06 ± 0.02 × 10^–3^ μm/s at [**1**·Zn^2+^] = 150 μM.

**Figure 4:**
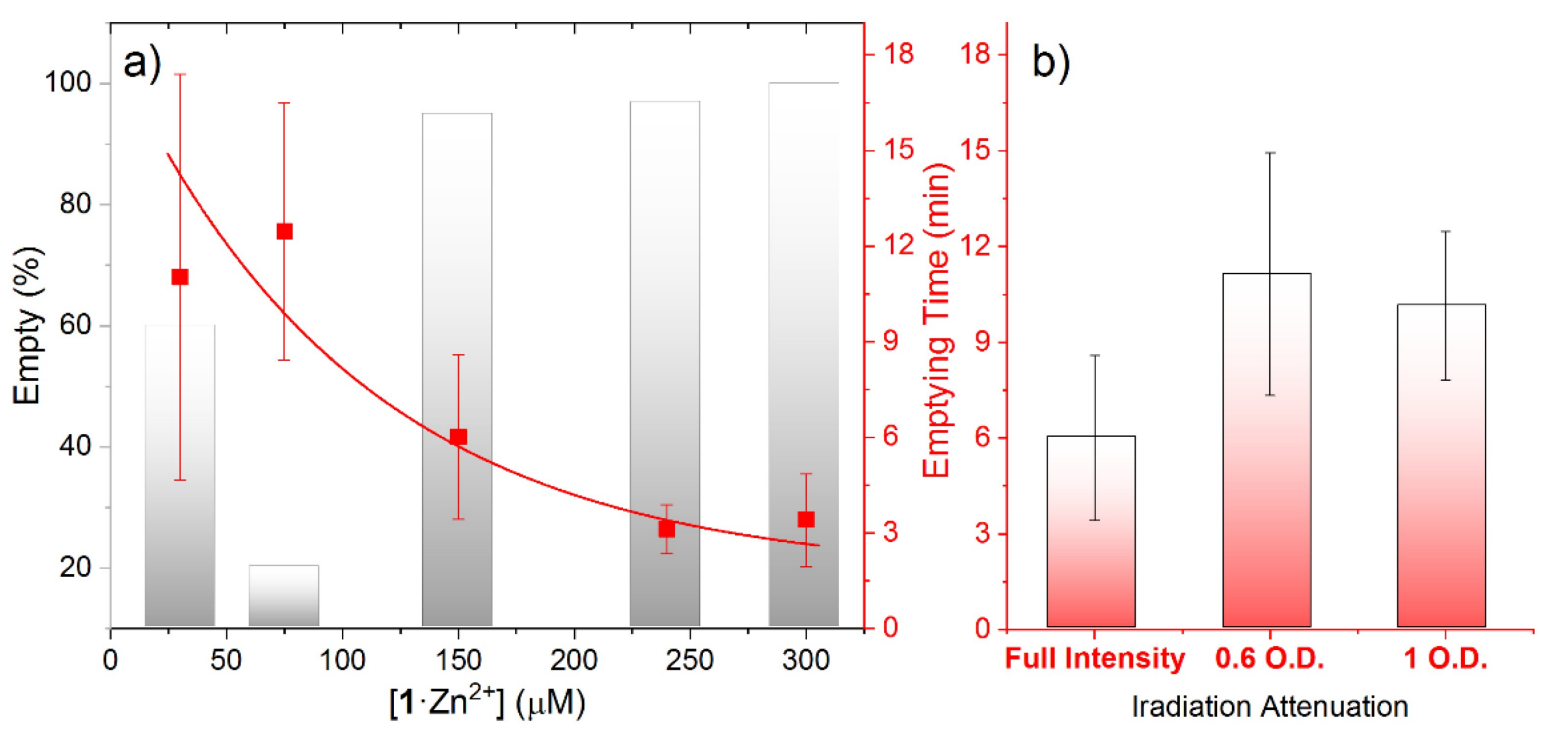
(**a**) Percentage of GUVs that release their cargo (gray bars) and their average emptying time (red squares) as a function of [**1**·Zn^2+^]. The red line is a visual guide; (**b**) average emptying times as a function of irradiation intensity for GUV populations when [**1**·Zn^2+^] = 150 µM. Error bars represent the standard deviation of the replicates.

We extended the investigation of the photoinduced release of sucrose to phospholipid GUVs incubated with several concentrations of **1**·Zn^2+^. The proportion of vesicles that released their contents and the average time of emptying could both be correlated to the concentration of **1**·Zn^2+^ in the GUV population (Figure 4a). Specifically, the percentage of vesicles that released their content increased with increasing [**1**·Zn^2+^], while the average release time decreased exponentially, reaching a plateau at POPC: **1**·Zn^2+^ 1:2. The analysis described for the study with [**1**·Zn^2+^] = 150 µM was applied to all other investigated concentrations, and the permeability varied in the interval of ~1–4 × 10^–3^ μm/s for 30 < [**1**·Zn^2+^] < 300 μM. Evidently, the number of pores and/or defects that favored the release of sucrose (or the intake of glucose) were proportional to the number of **1**·Zn^2+^ amphiphiles that the membrane was able to incorporate. We also observed that the photoswitchable molecule could induce bursting and cause the frequent explosion of GUVs possessing a diameter larger than 20–30 µm. This effect increased proportionally to the **1**·Zn^2+^ concentration. Lastly, no leaking of the encapsulated cargo was recorded in the absence of a UV light trigger over a period of 24 h.

Having demonstrated that the responsiveness of the GUVs could be regulated by the degree of **1**·Zn^2+^ incorporation, we proceeded to investigate irradiation intensity as an orthogonal approach for the control of release kinetics. Samples containing **1**·Zn^2+^ (150 µM) were irradiated with a UV light source together with neutral density filters possessing increasing optical densities. Figure 4b shows that irradiation at full intensity caused the disappearance of the contrast after an average of 6 min, whereas the attenuation of the light using neutral density filters with OD 0.6 or 1.0 resulted in a significant decrease in the rate of release to an average of 9–11 min. These results show that the cargo release characteristics of our GUVs could be tuned either during their formation by modifying the percentage incorporation of the photoswitchable amphiphile, or postsynthetically by changing the intensity of the light trigger.

## 4. Conclusions

In this work, we investigated the photoswitchable properties of an azobenzene-based amphiphile when incorporated into the membrane of mixed phospholipid/cholesterol giant vesicles. The *E*–*Z* photoisomerization induced by UV light triggered remarkable changes in the morphology of the vesicles, an effect that was dynamic and reversible. We interpreted these changes in terms of ADE theory, which provides rationale for the new equilibrium structures formed by the GUVs according to changes in the membrane surface area and in the volume of the inner lumen.

These findings were further exploited for the controlled tuning of membrane permeability. As a proof of concept, the photocontrollable release of sugars was characterized by means of phase-contrast and confocal microscopy. The initial limited permeability of sucrose could be increased to ~4 × 10^–3^ μm/s, allowing for complete release of the cargo within minutes. One key advantage of this modular multicomponent system is the flexibility in design that allows for easy modification of the proportions of the individual components. We demonstrated that changing the proportion of the photoswitchable molecule within the GUV could be used to tune the rate of cargo release. An alternative strategy to regulate drug release is to adjust the intensity of the light trigger. The demonstration that these orthogonal methods could be used in combination to fine-tune the membrane permeability of GUVs provides additional capabilities relevant to numerous medical and biomedical applications, such as targeted drug delivery and topical localized treatments. Studies on the cytotoxicity of the **1**·Zn^2+^ amphiphile are ongoing; however, previous studies on azobenzene-functionalized drugs and drug delivery systems, including light-responsive liposomes [67,68], self-assembled polymeric nanoparticles [69], and azo-based hydrogels [70], revealed the good biocompatibility of these engineered compounds. In particular, for cationic amphiphilic molecules with a similar chemical structure to that of **1**·Zn^2+^, the absence of cytotoxic effects of the photoswitchable compound [68,71] and of photophospholipid-based vesicle preparations [72] was assessed in the same concentration range as that of our working conditions. Lastly, the polar head group of **1**·Zn^2+^ (TACN) had none-to-negligible cytotoxic effects for concentrations up to 250 µM [73]. We are also confident that the use of POPC as the major component of the membrane, possibly doped with other natural phospholipids or sterols, can guarantee the effective biocompatibility of our photoresponsive vesicles. Moreover, the devised system could be used in combination with natural vesicles to engineer synthetic exosomes for nanomedicine applications [74], as previously reported for POPC liposomes fused with extracts of the standard cells lines ET3 and A549 [75], and with HUVEC-derived extracellular vesicles [76].

## Data Availability

Not applicable.
